# Real‐World Antithrombotic Management of Nonvalvular Atrial Fibrillation in Patients Undergoing Percutaneous Coronary Intervention in China: A Cross‐Sectional Study

**DOI:** 10.1155/cdr/5163410

**Published:** 2026-06-04

**Authors:** Jiwei Yu, Shuolin Liu, Run Du, Qinglan Tao, Jinzhou Zhu, Fenghua Ding, Ruiyan Zhang, Zhengbin Zhu

**Affiliations:** ^1^ Department of Cardiology, Rui Jin Hospital, Shanghai Jiao Tong University School of Medicine, Shanghai, China, shsmu.edu.cn; ^2^ Cardiovascular Research Institution, Shanghai Jiao Tong University School of Medicine, Shanghai, China, shsmu.edu.cn

**Keywords:** anticoagulation, antiplatelet, atrial fibrillation, percutaneous coronary intervention

## Abstract

Antithrombotic therapy in patients with nonvalvular atrial fibrillation (NVAF) undergoing percutaneous coronary intervention (PCI) is clinically challenging, with real‐world strategies often remaining inconsistent. This study analyzed 2020 annual data from the Shanghai Cardiac Intervention Quality Control Center, encompassing 23,115 patients who underwent PCI across 36 centers in Shanghai. Among these, 1306 (5.65%) patients with a confirmed diagnosis of NVAF were enrolled. Dual antiplatelet therapy (DAPT) was the most common antithrombotic regimen (584, 44.7%). Anticoagulation therapy was administered to only 478 (55.8%) of male patients with a CHA_2_DS_2_‐VASc score of ≥ 2 and 207 (55.6%) of female patients with a CHA_2_DS_2_‐VASc score of ≥ 3. Significant differences were observed across antithrombotic strategy groups regarding gender distribution, age, atrial fibrillation (AF) type, coronary artery disease type, and history of congestive heart failure (CHF), hypertension, stroke, or myocardial infarction (MI). Patients receiving oral anticoagulants (OACs) showed higher rates of persistent AF (53.2% vs. 33.9%, *p* < 0.001) and more frequent histories of CHF and stroke. Conversely, patients not receiving OAC therapy demonstrated a higher proportion of paroxysmal AF (66.1% vs. 46.8%, *p* < 0.001) and prior MI. A substantial gap persists between guideline recommendations and real‐world antithrombotic management in Chinese AF patients undergoing PCI. Key challenges include the underutilization of OAC therapy, a high prevalence of reduced‐dose anticoagulation, and a potential overreliance on DAPT. These findings highlight the urgent need to improve adherence to evidence‐based guidelines and conduct personalized risk–benefit assessments.

## 1. Introduction

Atrial fibrillation (AF), the most prevalent sustained cardiac arrhythmia worldwide, represents a growing global health burden. Epidemiological data indicate a steady rise in AF incidence over recent decades, with global prevalence surpassing 50 million cases by 2020 [1]. This trend is particularly pronounced in rapidly aging regions like Shanghai [2, 3]. Notably, 5%–15% of AF patients require percutaneous coronary intervention (PCI), necessitating optimized anticoagulation strategies to mitigate thromboembolic risks while balancing bleeding complications [4].

Contemporary antithrombotic management for patients with AF undergoing PCI has undergone significant paradigm shifts, driven by evolving evidence and updated international guidelines [1, 5]. Current recommendations advocate a time‐limited course of triple therapy (TT) (combining oral anticoagulants [OACs] with dual antiplatelet therapy [DAPT]) for post‐acute coronary syndrome (ACS) or PCI cases, with dual therapy (DT) (OAC plus a single antiplatelet agent) as an alternative regimen. However, individualized therapeutic decision‐making remains essential to achieve a delicate balance between thromboprophylaxis efficacy and hemorrhagic risk [6].

Real‐world implementation patterns may differ from guideline‐directed antithrombotic therapy. Guidelines provide standardized management frameworks; however, substantial geographical disparities in adherence have been documented. An Italian multicenter cohort study (73.6% anticoagulation rate in AF‐ACS/PCI patients) and Japanese data (86% direct oral anticoagulant [DOAC] utilization) contrast with Chinese single‐center findings (59.6% DAPT‐only use) [7–9]. A Thai cohort study revealed that 75.9% of AF patients with coronary artery disease (CAD) received anticoagulation therapy [10].

Moving beyond general utilization rates, recent domestic investigations have begun to dissect how specific clinical phenotypes and patient demographics influence treatment trajectories and long‐term outcomes in the Chinese population. Evidence suggests an increasing adoption of OACs and TT among patients with chronic coronary syndrome [11]. Furthermore, studies have demonstrated that females with AF and ACS are predisposed to a significantly higher incidence of long‐term adverse cardiovascular outcomes [12].

Despite these insights, clinical practice continues to grapple with the heightened bleeding risk inherent to Asian populations, leading to frequent DOAC underdosing [13, 14]. A recent study in China revealed that only 46.7% of patients received standard‐dose anticoagulation therapy [11]. Such deviations from evidence‐based protocols may be correlated with adverse clinical outcomes, including elevated thromboembolic and hemorrhagic events [15, 16]. However, most evidence is derived from specialized single‐center cohorts, which may not fully reflect the heterogeneous clinical practices across diverse healthcare tiers. Thus, city‐level multicenter data are essential to reveal real‐world antithrombotic patterns and inform region‐specific management strategies.

This cross‐sectional study aims to elucidate the real‐world application of antithrombotic therapy among patients with AF undergoing PCI in Shanghai, China, utilizing data from the Shanghai Cardiac Intervention Quality Control Center. Specifically, it seeks to examine variations in antithrombotic therapy regimens and evaluate the alignment between clinical practice and guideline recommendations, thereby providing insights into the state of balanced antithrombotic management in a real‐world Chinese setting.

## 2. Methods

### 2.1. Study Design and Patients

This cross‐sectional study analyzed annual registry data from the Shanghai Cardiac Intervention Quality Control Center, focusing on patients with nonvalvular atrial fibrillation (NVAF) who underwent PCI at 36 hospitals in Shanghai between January 1, 2020, and December 31, 2020. The Shanghai Cardiac Intervention Quality Control Center is a professional body authorized by the Shanghai Municipal Health Commission to oversee the quality of cardiac interventional procedures, including PCI. As data reporting is mandatory for all qualified hospitals as part of the city’s healthcare quality management and accreditation framework, the dataset encompasses all PCI patients treated within the study period across the city. Inclusion criteria were as follows: (1) aged > 18 years, (2) diagnosis of AF by electrocardiography, and (3) having undergone PCI within the specified timeframe. Exclusion criteria were as follows: (1) valvular AF and (2) incomplete medical records. This study was approved by the Ethics Committee of Ruijin Hospital (Ethics Approval No. 2026‐304) and conformed to the Declaration of Helsinki. Written informed consent for participation was waived in this study, as it was in accordance with national legislation and institutional requirements.

### 2.2. Definitions

The CHA_2_DS_2_‐VASc scores and HAS‐BLED scores were used to evaluate the risk of ischemic stroke and bleeding [17, 18]. High thromboembolic risk was defined as CHA_2_DS_2_‐VASc scores of ≥ 2 in males or ≥ 3 in females, while a high risk of bleeding was defined as a HAS‐BLED score of ≥ 3. PCI was defined as percutaneous transluminal coronary revascularization involving balloon angioplasty, drug‐eluting balloon angioplasty, or implantation of bare‐metal/drug‐eluting stents. Congestive heart failure (CHF) was diagnosed based on documented symptomatic heart failure and/or left ventricular ejection fraction (LVEF) of < 40%. Hypertension was defined as a documented clinical diagnosis or blood pressure measurements of ≥ 140/90 mmHg. History of stroke included ischemic or hemorrhagic cerebrovascular events confirmed by neuroimaging. Vascular diseases encompassed CAD with prior myocardial infarction (MI), acute myocardial infarction (AMI), or peripheral artery disease with confirmed arterial stenosis/occlusion. ACS comprised ST‐segment elevation myocardial infarction (STEMI), non‐ST‐segment elevation acute coronary syndrome (NSTE‐ACS), and unstable angina pectoris (UAP).

### 2.3. Antithrombotic Regimens

Antithrombotic regimens were evaluated based on the prescription of antithrombotic medications at discharge, including antiplatelet agents and OACs. Antiplatelet agents comprised aspirin, clopidogrel, ticagrelor, and cilostazol, while OACs included vitamin K antagonists (VKAs) and DOACs. Given the complexity of medication regimens in this patient population and to avoid an overly rigid classification of dose reductions as “inappropriate” based on conventional dose‐adjustment criteria, DOAC dosing was recorded observationally from discharge prescriptions. Based on the combination of prescribed medications, antithrombotic therapy was categorized into five regimens: single antiplatelet therapy (SAPT), DAPT, OAC monotherapy, TT, and DT.

Furthermore, TT and DT were further subclassified into DOAC‐based TT (DOAC+DAPT), VKA‐based TT (VKA+DAPT), DOAC‐based DT (DOAC+SAPT), and VKA‐based DT (VKA+SAPT).

### 2.4. Data Collection

Data on demographics, baseline comorbidities, and medication usage were collected by trained research personnel via a review of the hospitals’ electronic health records. These records included demographic profiles, previous medical histories, clinical characteristics, imaging results, and prescription medication use. All patients were assessed for their risk of thrombosis and bleeding using the CHA_2_DS_2_‐VASc and HAS‐BLED scoring systems. Individual CHA_2_DS_2_‐VASc and HAS‐BLED scores were calculated retrospectively based on baseline comorbidity data. To ensure data quality and integrity, a standardized quality control protocol was implemented throughout the study. Prior to study initiation, all site investigators underwent comprehensive training on the study protocol and standard operating procedures and utilized standardized Case Report Forms. All participant data provided by the centers were anonymized to protect confidentiality. Any identified data discrepancies were rigorously cross‐verified against original source documents.

### 2.5. Statistical Analysis

Descriptive statistics were used to summarize patient characteristics. Continuous variables were presented as mean ± standard deviation (SD) or as median and interquartile range (IQR) and were compared using Student’s *t*‐test or the Mann–Whitney *U*‐test between groups. Categorical variables were presented as frequencies with percentages, and group comparisons were made using the chi‐square test. Missing data for covariates were handled using multiple imputation by chained equations. The detailed proportion of missing data is provided in Table S1. All analyses were performed using the software packages SPSS (Version 25.0, IBM Corporation, New York, New York, United States) and GraphPad Prism 9.0. All statistical tests were two‐sided, and a value of *p* < 0.05 was considered significant.

## 3. Results

### 3.1. Demographic and Clinical Characteristics of the Study Population

This study identified 1306 (5.65%) patients with coexisting NVAF from a cohort of 23,115 individuals undergoing PCI. The median age was 73 years (IQR 68–80), with 385 (29.5%) being female. Significant gender distribution disparities were observed across different antithrombotic regimens: patients receiving DT demonstrated the highest proportion of females (110, 36.1%), while those receiving TT showed the lowest (78, 24.8%) (*p* = 0.009). Age stratification revealed a higher proportion of patients aged ≥ 75 years among those receiving DT (155, 50.8%) compared to those receiving TT (109, 34.7%) and DAPT (279, 47.8%) (*p* < 0.001).

Regarding clinical characteristics, paroxysmal AF accounted for 741 (56.7%). However, persistent/permanent AF was more prevalent among patients receiving DT, whereas paroxysmal AF predominated among those receiving DAPT (394, 67.5%). Analysis of CAD subtypes demonstrated that UAP was present in 588 (45.0%) patients, with ACS being more frequent among patients receiving DAPT (489, 83.7%). Common comorbidities included CHF (548, 42.0%), hypertension (1023, 78.3%), diabetes mellitus (508, 38.9%), prior MI (365, 27.9%), and history of stroke (263, 20.1%).

**Figure 1:**
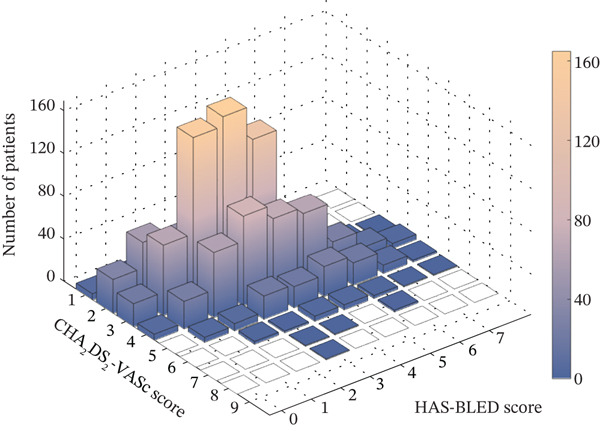
Distribution of CHA_2_DS_2_‐VASc and HAS‐BLED scores.

Risk stratification analysis indicated that 1240 (94.9%) patients had a CHA_2_DS_2_‐VASc score of ≥ 2, while 496 (38.0%) exhibited a HAS‐BLED score of ≥ 3. Figure 1 illustrates the distribution of CHA_2_DS_2_‐VASc and HAS‐BLED scores. Spearman’s correlation analysis revealed a positive association between thrombotic and bleeding risk scores (*r* = 0.454, *p* < 0.001).

Overall, 920 (70.4%) of patients received treatment at China Atrial Fibrillation Centers, with the DAPT group demonstrating a significantly lower proportion of patients treated at these centers (358, 61.3%) (*p* < 0.001). Left atrial appendage closure (LAAC) was performed in 100 (7.7%) of the study population. Among these, nine procedures were performed in patients with relatively lower risks of stroke and bleeding (eight male patients with a CHA_2_DS_2_‐VASc score of 1 and one male patient with a CHA_2_DS_2_‐VASc score of 2; all nine patients had a HAS‐BLED score of ≤ 2). Other demographic and clinical characteristics were comparable across different antithrombotic regimens (Table 1).

**Table 1:** Baseline characteristics of the study population ^∗^.

Variable	Total *N* = 1306	TT, *n* (%) *N* = 314	DT, *n* (%) *N* = 305	DAPT, *n* (%) *N* = 584	OAC, *n* (%) *N* = 100	SAPT, *n* (%) *N* = 3	*p* value
*Demographic characteristics*							
Sex (female sex), *n* (%)	385 (29.5%)	78 (24.8%)	110 (36.1%)	172 (29.5%)	25 (25.0%)	0 (0.0%)	0.019
Age at diagnosis, median (IQR)	73 (68–80)	72 (67–79)	74 (69–80)	73 (67–81)	72.5 (67–78)	71 (69.5–71)	0.001
< 65, *n* (%)	205 (15.7%)	57 (18.2%)	35 (11.5%)	98 (16.8%)	15 (15.0%)	0 (0.0%)	< 0.001
65–74, *n* (%)	515 (39.4%)	148 (47.1%)	115 (37.7%)	207 (35.4%)	42 (42.0%)	3 (100.0%)	
≥ 75, *n* (%)	586 (44.9%)	109 (34.7%)	155 (50.8%)	279 (47.8%)	43 (43.0%)	0 (0.0%)	
BMI, median (IQR)	24.6 (22.6–26.1)	24.3 (22.2–26.6)	24.8 (23.0–26.2)	24.6 (22.5–26.4)	24.6 (23.2–25.5)	25.9 (25.7–26.8)	0.237
*AF type,* *n* *(%)*							
Paroxysmal AF	741 (56.7%)	167 (53.2%)	126 (41.3%)	394 (67.5%)	54 (54.0%)	0 (0.0%)	< 0.001
Persistent/permanent AF	565 (43.3%)	151 (48.1%)	181 (59.3%)	198 (33.9%)	46 (46.0%)	3 (100.0%)	< 0.001
*CAD type,* *n* *(%)*							
SAP	326 (25.0%)	70 (22.3%)	93 (30.5%)	95 (16.3%)	65 (65.0%)	3 (100.0%)	< 0.001
UAP	588 (45.0%)	159 (50.6%)	151 (49.5%)	251 (43.0%)	27 (27.0%)	0 (0.0%)	
NSTE‐ACS	175 (13.4%)	40 (12.7%)	28 (9.2%)	140 (24.0%)	4 (4.0%)	0 (0.0%)	
STEMI	217 (16.6%)	45 (14.3%)	33 (10.8%)	98 (16.8%)	4 (4.0%)	0 (0.0%)	
*Medical history,* *n* *(%)*							
CHF	548 (42%)	140 (44.6%)	148 (48.5%)	208 (35.6%)	51 (51.0%)	1 (33.3%)	< 0.001
NYHA I	17 (1.3%)	9 (2.9%)	1 (0.3%)	7 (1.2%)	0 (0.0%)	0 (0.0%)	< 0.001
NYHA II	286 (21.9%)	70 (22.3%)	83 (27.2%)	106 (18.2%)	27 (27.0%)	0 (0.0%)	
NYHA III	202 (15.5%)	50 (15.9%)	56 (18.4%)	74 (12.7%)	21 (21.0%)	1 (33.3%)	
NYHA IV	43 (3.3%)	11 (3.5%)	8 (2.6%)	21 (3.6%)	3 (3.0%)	0 (0.0%)	
Hypertension	1023 (78.3%)	236 (75.2%)	254 (83.3%)	443 (75.9%)	87 (87.0%)	3 (100.0%)	0.009
Hyperlipemia	121 (9.3%)	29 (9.2%)	22 (7.2%)	60 (10.3%)	10 (10.0%)	0 (0.0%)	0.626
DM	508 (38.9%)	123 (39.2%)	121 (39.7%)	221 (37.8%)	41 (41.0%)	2 (66.7%)	0.823
Stroke	263 (20.1%)	103 (32.8%)	76 (24.9%)	198 (33.9%)	22 (22.0%)	0 (0.0%)	< 0.001
Ischemic	251 (19.2%)	71 (22.6%)	74 (24.3%)	84 (14.4%)	22 (22.0%)	0 (0.0%)	0.017
Hemorrhagic	10 (0.8%)	4 (1.3%)	4 (1.3%)	2 (0.3%)	0 (0.0%)	0 (0.0%)	
Ischemic+hemorrhagic	2 (0.1%)	0 (0.0%)	0 (0.0%)	2 (0.3%)	0 (0.0%)	0 (0.0%)	
Prior MI	365 (27.9%)	77 (24.5%)	61 (20.1%)	205 (35.2%)	21 (21.0%)	1 (33.3%)	< 0.001
Prior bleeding	36 (2.8%)	7 (2.2%)	9 (2.9%)	16 (2.7%)	4 (4.0%)	0 (0.0%)	0.905
Renal disease (Cr > 2.26 mg/dL)	84 (6.4%)	15 (4.8%)	16 (5.2%)	45 (7.7%)	7 (7.0%)	1 (33.3%)	0.116
Current smoker	396 (30.3%)	103 (32.8%)	76 (24.9%)	198 (33.9%)	17 (17.0%)	2 (66.7%)	< 0.001
Current drinker	183 (14%)	60 (19.1%)	41 (13.4%)	65 (11.1%)	17 (17.0%)	0 (0.0%)	0.017
*Echocardiographic, median (IQR)*							
LVEF	59 (50–64)	58 (49–62)	60 (52–64)	59 (51–64)	63 (55–65)	58 (52–59)	< 0.001
LA	43 (39–48)	43 (38–48)	44 (40–48)	42 (39–46)	43 (40–48)	49 (48.5–50.5)	0.30
PASP	34 (25–40)	32 (25–40)	35 (28–40)	33 (26–41)	25 (23–25)	20 (10–23)	0.052
*Clinical risk score*							
CHA_2_DS_2_‐VASc score, mean (SD)	4.06 (1.65)	4.00 (1.60)	4.36 (1.73)	3.92 (1.60)	4.16 (1.81)	3.33 (0.58)	0.002
1, *n* (%)	66 (5.1%)	13 (4.1%)	10 (3.3%)	37 (6.3%)	6 (6.0%)	0 (0.0%)	0.302
2, *n* (%)	162 (12.4%)	45 (14.3%)	36 (11.8%)	69 (11.8%)	12 (12.0%)	0 (0.0%)	
3, *n* (%)	281 (21.5%)	69 (22.0%)	51 (16.7%)	137 (23.5%)	22 (22.0%)	2 (66.7%)	
4, *n* (%)	304 (23.3%)	74 (23.6%)	69 (22.6%)	141 (24.1%)	19 (19.0%)	1 (33.3%)	
5, *n* (%)	246 (18.8%)	52 (16.6%)	67 (22.0%)	109 (18.7%)	18 (18.0%)	0 (0.0%)	
6, *n* (%)	145 (11.1%)	41 (13.1%)	37 (12.1%)	54 (9.2%)	13 (13.0%)	0 (0.0%)	
7, *n* (%)	69 (5.3%)	16 (5.1%)	22 (7.2%)	26 (4.5%)	5 (5.0%)	0 (0.0%)	
8, *n* (%)	24 (1.8%)	3 (1.0%)	8 (2.6%)	9 (1.5%)	4 (4.0%)	0 (0.0%)	
9, *n* (%)	9 (0.7%)	1 (0.3%)	5 (1.6%)	2 (0.3%)	1 (1.0%)	0 (0.0%)	
HAS‐BLED score, mean (SD)		2.44 (0.96)	2.43 (1.00)	2.32 (0.91)	1.76 (0.98)	2.00 (1.73)	< 0.001
0, *n* (%)	25 (1.9%)	5 (1.6%)	6 (2.0%)	5 (0.9%)	9 (9.0%)	0 (0.0%)	< 0.001
1, *n* (%)	182 (13.9%)	31 (9.9%)	35 (11.5%)	83 (14.2%)	31 (31.0%)	2 (66.7%)	
2, *n* (%)	603 (46.2%)	146 (46.5%)	132 (43.3%)	286 (49.0%)	39 (39.0%)	0 (0.0%)	
3, *n* (%)	358 (27.4%)	94 (29.9%)	97 (31.8%)	150 (25.7%)	17 (17.0%)	0 (0.0%)	
4, *n* (%)	113 (8.7%)	30 (9.6%)	27 (8.9%)	51 (8.7%)	4 (4.0%)	1 (33.3%)	
5, *n* (%)	20 (1.5%)	6 (1.9%)	5 (1.6%)	9 (1.5%)	0 (0.0%)	0 (0.0%)	
6, *n* (%)	4 (0.3%)	2 (0.6%)	2 (0.7%)	0 (0.0%)	0 (0.0%)	0 (0.0%)	
7, *n* (%)	1 (0.1%)	0 (0.0%)	1 (0.3%)	0 (0.0%)	0 (0.0%)	0 (0.0%)	
*Medical institution profile*							
Treated in China Atrial Fibrillation Centers, *n* (%)	920 (70.4%)	230 (73.2%)	234 (76.7%)	358 (61.3%)	95 (95.0%)	3 (100.0%)	< 0.001
PCI procedure volume, median (IQR)	1213 (870–1811)	1213 (619–1913)	1213 (870–1974)	1213 (619–1921)	1213 (1213–1213)	1213 (1213–1567)	< 0.001
*Left atrial appendage closure,* *n* *(%)*	100 (7.7%)	20 (6.4%)	39 (12.8%)	38 (6.5%)	3 (3.0%)	3 (100.0%)	0.002

Abbreviations: AF, atrial fibrillation; BMI, body mass index; CAD, coronary artery disease; CHF, congestive heart failure; Cr, creatinine; DAPT, dual antiplatelet therapy; DM, diabetes mellitus; DT, dual therapy; IQR, interquartile range; LA, left atria; LVEF, left ventricular ejection fraction; NSTE‐ACS, non‐ST‐segment elevation acute coronary syndrome; NYHA, New York Heart Association; OAC, oral anticoagulant; PASP, pulmonary arterial systolic pressure; PCI, percutaneous coronary intervention; SAP, stable angina pectoris; SAPT, single antiplatelet therapy; SD, standard deviation; STEMI, ST‐segment elevation myocardial infarction; TT, triple therapy; UAP, unstable angina pectoris.

^∗^Comparisons (*p* values) were made among the four main antithrombotic strategy groups (TT, DT, DAPT, and OAC). Three patients who received SAPT are listed for completeness but were excluded from statistical comparisons due to the distinct clinical profile and small sample size.

Comparative analysis by gender revealed significant age differences, with male patients demonstrating a lower median age (72 years, IQR 66–78) compared to females (77 years, IQR 72–83; *p* < 0.001). Male patients exhibited higher rates of prior MI (29.9% vs. 23.6%, *p* = 0.021) and lower LVEF (55.3 ± 13.3 vs. 58.3 ± 12.5, *p* < 0.001). Conversely, female patients had significantly higher rates of hypertension (82.1% vs. 76.8%, *p* = 0.034) and CHA_2_DS_2_‐VASc scores (5.0 ± 1.57 vs. 3.67 ± 1.53, *p* < 0.001) (Table S2).

### 3.2. Status of Antithrombotic Therapy

**Figure 2:**
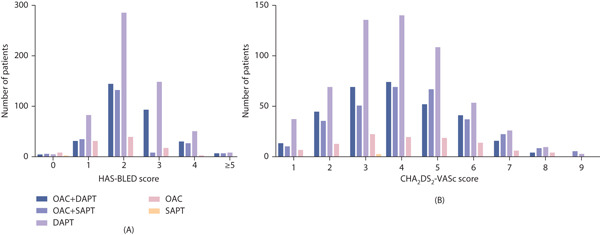
(A) Antithrombotic therapy distribution stratified by HAS‐BLED score. (B) Antithrombotic therapy distribution stratified by CHA_2_DS_2_‐VASc score. Abbreviations: DAPT, dual antiplatelet therapy; OAC, oral anticoagulant; SAPT, single antiplatelet therapy.

**Figure 3:**
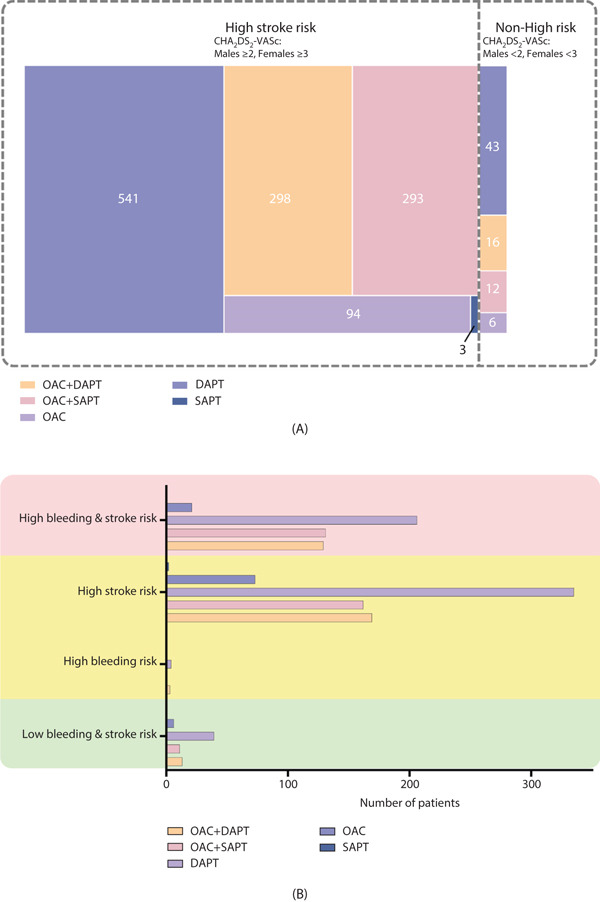
(A) Detailed distribution of treatment regimens among patients categorized as having high or non‐high stroke risk, defined as CHA_2_DS_2_‐VASc scores of ≥ 2 for males and ≥ 3 for females. (B) Antithrombotic strategies by risk group. Distribution of prescribed regimens among patients stratified by concurrently assessed stroke and bleeding risk. High bleeding risk: HAS‐BLED of ≥ 3. High stroke risk: CHA_2_DS_2_‐VASc scores of ≥ 2 for males and ≥ 3 for females. Low bleeding risk: HAS‐BLED of < 3. Low stroke risk: CHA_2_DS_2_‐VASc scores of < 2 for males and < 3 for females. Abbreviations: DAPT, dual antiplatelet therapy; OAC, oral anticoagulant; SAPT, single antiplatelet therapy.

Table 2 details the discharge medication regimens prescribed to patients who had NVAF and underwent PCI. These data were collected at a median of 3 days after the procedure (IQR 2–5 days). Figure 2 illustrates the distribution of antithrombotic therapy stratified by both CHA_2_DS_2_‐VASc and HAS‐BLED scores. Anticoagulation was administered to 478 (55.8%) of males with a CHA_2_DS_2_‐VASc score of ≥ 2 and 207 (55.6%) of females with a score of ≥ 3. Based on these scores, patients were further stratified into high‐ and non‐high‐stroke‐risk groups. Figure 3A shows the distribution of the different antithrombotic regimens. Figure 3B shows the distribution of prescribed regimens among patients stratified by concurrently assessed stroke and bleeding risk.

**Table 2:** Antithrombotic therapy and drug usage of patients with AF who underwent PCI.

Detailed antithrombotic therapy regimens, *n* (%)	
OAC+DAPT	314 (24.0%)
DOAC+DAPT	265 (20.3%)
Rivaroxaban+DAPT	231 (17.7%)
+ASA+clopidogrel	200 (15.3%)
+ASA+ticagrelor	23 (1.8%)
+Cilostazol+clopidogrel	7 (0.5%)
+Cilostazol+ticagrelor	1 (0.1%)
Dabigatran+DAPT	34 (2.6%)
+ASA+clopidogrel	30 (2.3%)
+ASA+ticagrelor	3 (0.2%)
+Cilostazol+clopidogrel	1 (0.1%)
VKA+DAPT	49 (3.7%)
+ASA+clopidogrel	42 (3.2%)
+ASA+ticagrelor	6 (0.5%)
+Cilostazol+clopidogrel	1 (0.1%)
OAC+SAPT	305 (23.4%)
DOAC+SAPT	282 (21.6%)
Rivaroxaban+SAPT	247 (18.9%)
+Clopidogrel	204 (15.6%)
+ASA	22 (1.7%)
+Ticagrelor	21 (1.6%)
Dabigatran+SAPT	35 (2.7%)
+Clopidogrel	29 (2.2%)
+ASA	3 (0.2%)
+Ticagrelor	3 (0.2%)
VKA+SAPT	23 (1.8%)
+Clopidogrel	16 (1.2%)
+ASA	5 (0.4%)
+Ticagrelor	2 (0.2%)
DAPT	584 (44.7%)
ASA+clopidogrel	330 (25.3%)
ASA+ticagrelor	230 (17.6%)
Cilostazol+clopidogrel	21 (1.6%)
Cilostazol+ticagrelor	3 (0.2%)
OAC	100 (7.7%)
DOAC	85 (6.6%)
Rivaroxaban	74 (5.7%)
Dabigatran	11 (0.8%)
VKA	15 (1.1%)
SAPT	3 (0.2%)

Abbreviations: ASA, acetylsalicylic acid; DAPT, dual antiplatelet therapy; DOAC, direct‐acting oral anticoagulant; OAC, oral anticoagulant; SAPT, single antiplatelet therapy; VKA, vitamin K antagonist.

TT was administered to 314 (24.0%) of patients, with males receiving TT more frequently than females (25.6% vs. 20.3%, *p* = 0.019). DOAC‐based TT regimens predominated, accounting for 265 (20.3%) of the total cohort. DT was utilized in 305 (23.4%) patients, which showed a higher adoption rate in females (28.6% vs. 21.2%, *p* = 0.019), primarily through DOAC+SAPT combinations (21.6%, 282/1306).

Rivaroxaban‐based regimens constituted the predominant therapeutic approach (552, 76.8%), whereas dabigatran utilization was limited to 80 (11.1%). Among rivaroxaban recipients, dosing regimens demonstrated notable variation: 256 (46.4%) received reduced dosing (5 mg qd or 2.5 mg bid), 168 (30.4%) received 15 mg qd, and 10 (1.8%) received 20 mg qd. All dabigatran‐treated patients received standardized dosing of 110 mg bid. For warfarin users, 42 (45.6%) of the 92 patients achieved therapeutic in‐hospital international normalized ratio levels within the 2–3 range (Table S3).

**Figure 4:**
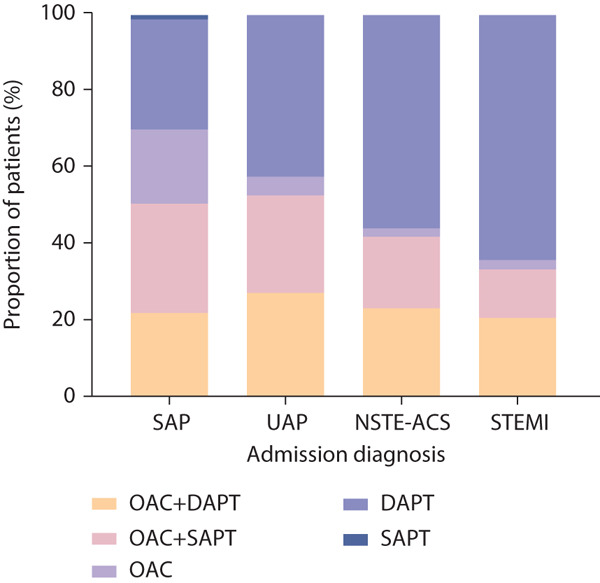
Antithrombotic regimens stratified by CAD diagnosis. Abbreviations: DAPT, dual antiplatelet therapy; NSTE‐ACS, non‐ST‐segment elevation acute coronary syndrome; OAC, oral anticoagulant; SAP, stable angina pectoris; SAPT, single antiplatelet therapy; STEMI, ST‐segment elevation myocardial infarction; UAP, unstable angina pectoris.

Significant therapeutic strategy variations were observed across CAD subtypes (Figure 4). The proportions of patients receiving DT across different CAD subtypes were 93 (28.5%) in SAP, 151 (25.7%) in UAP, and 28 (12.9%) in STEMI patients. DAPT emerged as the predominant therapeutic approach in STEMI management (140, 64.5%) (*p* < 0.001 compared with other subtypes). OAC monotherapy demonstrated preferential application in SAP patients.

### 3.3. Subgroup Analysis by Antithrombotic Therapy

We analyzed clinical characteristics in a high‐stroke‐risk population, defined as CHA_2_DS_2_‐VASc scores of ≥ 2 for males and ≥ 3 for females. Based on stroke prevention strategies, patients were stratified into three therapeutic groups: the OAC+ group (receiving OACs; *N* = 628), the OAC− group (not receiving OACs; *N* = 540), and the LAAC group (*N* = 91). Baseline characteristics are systematically compared in Table 3.

**Table 3:** Clinical characteristics of the study population stratified by anticoagulation approach.

Variable	OACs+ *N* = 628	OACs− *N* = 510	LAAC ^∗^ *N* = 91	*p* value ^∗∗^
*Demographic characteristics*				
Sex (female sex), *n* (%)	188 (29.9%)	150 (29.4%)	34 (37.4%)	0.304
Age at diagnosis, median (IQR)	73 (68–79)	75 (69–81)	76 (68–82)	0.05
< 65, *n* (%)	85 (13.5%)	65 (12.7%)	7 (7.7%)	0.118
65–74, *n* (%)	265 (42.2%)	189 (37.1%)	34 (37.4%)	
≥ 75, *n* (%)	278 (44.3%)	256 (50.2%)	50 (54.9%)	
BMI, median (IQR)	24.7 (22.8–26.3)	24.6 (22.5–26.6)	24.5 (22.1–26.1)	0.303
*AF type,* *n* *(%)*				
Paroxysmal AF	294 (46.8%)	337 (66.1%)	41 (45.1%)	< 0.001
Persistent/permanent AF	334 (53.2%)	173 (33.9%)	50 (54.9%)	
*CAD type,* *n* *(%)*				
SAP	196 (31.2%)	84 (16.5%)	24 (26.4%)	< 0.001
UAP	290 (46.2%)	211 (41.4%)	53 (58.2%)	
NSTE‐ACS	68 (10.8%)	91 (17.8%)	9 (9.9%)	
STEMI	74 (11.8%)	124 (24.3%)	5 (5.5%)	
*Medical history,* *n* *(%)*				
CHF	308 (49.0%)	193 (37.8%)	40 (44.0%)	< 0.001
NYHA I	10 (1.6%)	6 (1.2%)	0 (0.0%)	0.011
NYHA II	162 (25.8%)	98 (19.2%)	25 (27.5%)	
NYHA III	115 (18.3%)	69 (13.5%)	14 (15.4%)	
NYHA IV	21 (3.3%)	20 (3.9%)	1 (1.1%)	
Hypertension	520 (82.8%)	401 (78.6%)	77 (84.6%)	0.138
Hyperlipemia	54 (8.6%)	52 (10.2%)	7 (7.7%)	0.570
DM	262 (41.7%)	209 (41.0%)	34 (37.4%)	0.731
Stroke	152 (24.2%)	81 (15.9%)	30 (33.0%)	< 0.001
Ischemic	147 (23.4%)	77 (15.1%)	27 (29.7%)	< 0.001
Hemorrhagic	5 (0.8%)	2 (0.4%)	3 (3.3%)	
Ischemic+hemorrhagic	0 (0.0%)	2 (0.4%)	0 (0.0%)	
Prior MI	152 (24.2%)	194 (38.1%)	13 (14.3%)	< 0.001
Prior bleeding	11 (1.8%)	14 (2.7%)	9 (9.9%)	< 0.001
Renal disease (Cr > 2.26 mg/dL)	32 (5.1%)	41 (8.1%)	4 (4.4%)	0.092
Current smoker	168 (26.8%)	169 (33.1%)	23 (25.3%)	0.043
Current drinker	90 (14.3%)	40 (7.8%)	47 (51.6%)	< 0.001
*Echocardiographic, median (IQR)*				
LVEF	59 (49–65)	58 (50–63)	62 (55–65)	0.026
LA	43 (39–48)	42 (38–46)	44 (39–48)	0.007
PASP	35 (25–42)	34 (26–41)	35 (30–38)	0.731
*Clinical risk score*				
CHA_2_DS_2_‐VASc score, mean (SD)	4.32 (1.58)	4.15 (1.46)	4.23 (1.53)	0.157
2, *n* (%)	80 (12.7%)	60 (11.8%)	11 (12.1%)	0.891
3, *n* (%)	132 (21.0%)	127 (24.9%)	22 (24.2%)	
4, *n* (%)	148 (23.6%)	134 (26.3%)	22 (24.2%)	
5, *n* (%)	128 (20.4%)	101 (19.8%)	17 (18.7%)	
6, *n* (%)	81 (12.9%)	52 (10.2%)	12 (13.2%)	
7, *n* (%)	39 (6.2%)	25 (4.9%)	5 (5.5%)	
8, *n* (%)	14 (2.2%)	9 (1.8%)	1 (1.1%)	
9, *n* (%)	6 (1.0%)	2 (0.4%)	1 (1.1%)	
HAS‐BLED score, mean (SD)	2.34 (0.94)	2.35 (0.88)	2.92 (1.18)	< 0.001
0, *n* (%)	15 (2.4%)	3 (0.6%)	0 (0.0%)	< 0.001
1, *n* (%)	78 (12.4%)	60 (11.8%)	7 (7.7%)	
2, *n* (%)	287 (45.7%)	262 (51.4%)	29 (31.9%)	
3, *n* (%)	188 (29.9%)	133 (26.1%)	30 (33.0%)	
4, *n* (%)	50 (8.0%)	43 (8.4%)	19 (20.9%)	
5, *n* (%)	9 (1.4%)	9 (1.8%)	2 (2.2%)	
6, *n* (%)	1 (0.2%)	0 (0.0%)	3 (3.3%)	
7, *n* (%)	0 (0.0%)	0 (0.0%)	1 (1.1%)	
≥ 3, *n* (%)	248 (39.5%)	185 (36.3%)	55 (64.4%)	< 0.001
*Medical institution profile*				
Treated in China Atrial Fibrillation Centers, *n* (%)	500 (79.6%)	316 (62.0%)	48 (52.7%)	< 0.001
PCI procedure volume, median (IQR)	1213 (729–1921)	1213 (619–1921)	1213 (755–1391)	0.123

Abbreviations: AF, atrial fibrillation; BMI, body mass index; CAD, coronary artery disease; CHF, congestive heart failure; Cr, creatinine; DM, diabetes mellitus; LA, left atria; LAAC, left atrial appendage closure; LVEF, left ventricular ejection fraction; NSTE‐ACS, non‐ST‐segment elevation acute coronary syndrome; NYHA, New York Heart Association; OACs+, oral anticoagulants prescribed; OACs−, oral anticoagulants not prescribed; PASP, pulmonary arterial systolic pressure; PCI, percutaneous coronary intervention; SAP, stable angina pectoris; STEMI, ST‐segment elevation myocardial infarction; UAP, unstable angina pectoris.

^∗^A total of 100 patients underwent LAAC. For the purpose of this outcome analysis, the LAAC group is strictly defined to include only the 91 patients who received on‐label LAAC procedures.

^∗∗^Comparisons were made among treatment groups.

No significant intergroup differences existed in gender distribution (*p* > 0.05), though marked disparities emerged in AF classification: the OAC− group demonstrated higher paroxysmal AF prevalence (66.1% vs. 46.8% in the OAC+ group vs. 45.1% in the LAAC group; *p* < 0.001), while persistent/permanent AF predominated in the OAC+ and LAAC groups. Significant CAD subtype stratification revealed higher STEMI rates in the OAC− group (24.3% vs. 11.8% in the OAC+ group vs. 5.5% in the LAAC group; *p* < 0.05). CHF was more frequent in the OAC+ group than in the OAC− group (49.0% vs. 37.8%; *p* < 0.05), whereas prior stroke history was significantly lower in the OAC− group (15.9% vs. 24.2% in the OAC+ group vs. 33.0% in the LAAC group; *p* < 0.001).

Conversely, MI history was elevated in the OAC− group (38.1% vs. 24.2% in the OAC+ group vs. 44.0% in the LAAC group; *p* < 0.001), and prior bleeding history was more common in the LAAC group (9.9% vs. 1.8% in the OAC+ group vs. 2.7% in the OAC− group; *p* < 0.001). Notably, the LAAC group demonstrated significantly higher HAS‐BLED scores, with 55 (60.4%) scoring ≥ 3, whereas no significant difference was observed between the OAC+ and OAC− groups. In contrast, CHA_2_DS_2_‐VASc scores showed no significant variation across all three groups (*p* = 0.66). OAC+ patients were more frequently managed at China Atrial Fibrillation Centers (79.6% vs. 62.0% for the OAC− group vs. 52.7% for the LAAC group; *p* < 0.001).

## 4. Discussion

For patients with AF comorbid with PCI, balancing ischemic and bleeding risks remains a central challenge in clinical practice. The international guidelines have established consensus recommendations: short‐term triple antithrombotic therapy transitioning to DT in NVAF patients post‐ACS/PCI, with DOACs preferred over VKAs and clopidogrel as the recommended P2Y12 inhibitor [5, 19–21]. However, this cross‐sectional study suggests potential discrepancies between these recommendations and clinical practice in China.

Our cross‐sectional study reveals critical disparities between contemporary antithrombotic strategies and consensus recommendations for NVAF patients undergoing PCI, exposing fundamental challenges in thromboembolic risk management. The observed 5.65% AF prevalence among Shanghai’s PCI population—characterized by advanced age (median 73 years), elevated thrombotic burden (1240 [94.9%] with CHA_2_DS_2_‐VASc of ≥ 2), and heightened bleeding susceptibility (496 [38.0%] with HAS‐BLED of ≥ 3)—underscores the imperative for precision medicine in this cohort.

The observed shortfall in anticoagulation therapy among high‐thrombotic‐risk patients highlights a systemic underuse of risk stratification tools. Specifically, only 478 (55.8%) of men with a CHA_2_DS_2_‐VASc score of ≥ 2 and 207 (55.6%) of women with a score of ≥ 3 received anticoagulation therapy. Meanwhile, DAPT was used in 584 (44.7%) of discharges; others received SAPT or OAC monotherapy. This pattern is consistent with studies from Beijing (59.8% DAPT use) [9], South Korea (49.8% DAPT use) [22], and Denmark (< 50% on DT/TT) [23], suggesting a potential common trend across different settings. This pattern could be influenced by vigilance and concern regarding bleeding events. In the study population, 36 patients had a prior history of bleeding (including 12 cases of hemorrhagic stroke and 24 cases of gastrointestinal bleeding). Among these patients, 16 (44.4%) received OAC therapy, while 26 (72.2%) underwent LAAC. The preference for DAPT after PCI may be attributed to concerns about bleeding risks associated with DT/TT, a practice that persists despite evidence demonstrating acceptable bleeding risks between DOAC‐based trials. This gap is further compounded by the correlation between CHA_2_DS_2_‐VASc and HAS‐BLED scores (*r* = 0.454, *p* < 0.001), which complicates the risk–benefit calculus.

The observed prevalence of reduced‐dose DOACs and the deviation in P2Y12 inhibitor selection suggest a complex clinical trade‐off. In this study, the usage rate of clopidogrel was 67.5% (882/1306), lower than the 88%–94.4% reported in previous randomized controlled trials [24]. Ticagrelor was prescribed more frequently to patients with ACS than to those with stable angina pectoris (SAP) (25.7% vs. 12.6%, *p* < 0.001), likely due to the need for stronger antiplatelet effects, which may have driven physicians to prefer DAPT over TT/DT and increased anticoagulation avoidance. Unlike AF monotherapy, which has well‐defined dose‐reduction criteria, there is currently a lack of universally standardized guidelines for DOAC dosing when combined with antiplatelet agents post‐PCI. In the absence of such consensus, many clinicians empirically opt for lower doses as a precautionary strategy to mitigate the heightened bleeding risk associated with combined therapy. While this modification aims to balance safety and efficacy, evidence from multicenter observational studies suggests that patients on off‐label low‐dose DOACs may experience a higher incidence of stroke compared to those on standard doses [25]. These findings highlight the urgent need for more granular evidence to guide individualized antithrombotic optimization in this high‐risk population.

Interventions aimed at improving anticoagulation prescribing patterns in AF can be effective, though outcomes vary across intervention types. Educational interventions targeting healthcare professionals have proven to be one effective approach. A cluster trial demonstrated that such education led to higher rates of anticoagulant prescription compared with usual care (odds ratio 3.28, 95% CI 1.67–6.44) and a lower incidence of stroke in the intervention group (hazard ratio 0.48, 95% CI 0.23–0.99) [26]. Additionally, previous studies have confirmed that targeted patient education and electronic risk assessment or decision support systems can also improve anticoagulation prescribing patterns in AF patients [27].

Stratified analyses identified associations between patient characteristics and treatment choices, suggesting patterns that may reflect how clinicians assess and respond to risks. The OAC+ group was characterized by a clinical profile often associated with a thrombosis‐oriented decision‐making approach, including mainly persistent/permanent AF, a higher rate of prior stroke, and more prior heart failure. Evidence suggests that a higher AF burden is associated with a significantly increased risk of stroke [28], which could make intensified anticoagulation therapy a more considered option for these patients. The LAAC group demonstrated characteristics of elevated bleeding and thromboembolic risk, evidenced by notably higher rates of prior stroke history, prior bleeding history, and elevated HAS‐BLED scores. It is important to note that the HAS‐BLED score identifies modifiable bleeding risk factors, not contraindications to anticoagulation [5]. For these high‐risk patients, personalized approaches, such as incorporating LAAC, may improve the risk–benefit balance. However, the observed utilization rate of LAAC contrasts with its demonstrated efficacy in high‐bleeding‐risk populations. As a nonpharmacological alternative to oral anticoagulation for AF, LAAC has shown similar efficacy and safety compared with VKAs or DOACs [29].

The OAC− group was characterized by paroxysmal AF, STEMI, and a history of MI. This may reflect that ACS events drive clinicians to consider aggressive antiplatelet regimens. In STEMI patients, DAPT usage is as high as 64.5% (140/217), with 57.1% (80/140) on a ticagrelor‐containing DAPT regimen, which may delay OAC reintroduction. A recent study showed that in‐hospital anticoagulation for STEMI patients with new‐onset AF is associated with reduced all‐cause mortality but with similar major bleeding rates [30].

The China Atrial Fibrillation Center serves as a crucial driver in advancing the management of AF, having established a comprehensive set of evaluation standards for healthcare institutions across the full spectrum of AF care. These standards encompass AF screening, standardized outpatient and inpatient management, procedures such as catheter ablation and LAAC, postdischarge follow‐up, and a tiered diagnosis‐and‐treatment framework spanning different levels of medical facilities [31–33]. In our study, 920 (70.4%) of patients were treated at China Atrial Fibrillation Centers, and the OAC+ group had a higher proportion of patients treated at these centers. This observation suggests that standardized AF centers are more likely to implement guideline‐recommended multidisciplinary care. However, the widespread underanticoagulation and dosage deviations indicate that center‐based stratification alone may be insufficient to address the observed practice variations. Looking ahead, more real‐world evidence is needed. Future studies should monitor clinical outcomes of patients receiving non‐guideline‐adherent dosing and explore best practices for the Chinese population.

## 5. Conclusion

This cross‐sectional study reveals significant disparities between guideline recommendations and real‐world antithrombotic management in Chinese AF patients post‐PCI. Key concerns include the underutilization of anticoagulation, overreliance on DAPT, and inappropriate DOAC underdosing. These findings underscore an urgent need for improved adherence to evidence‐based guidelines and personalized risk–benefit assessments.

## 6. Limitations

This study has several limitations that should be considered when interpreting its findings. First, as a cross‐sectional observational study based on retrospective data from a single year, it can only describe associations and practice patterns at discharge rather than establish causality or evaluate long‐term clinical outcomes such as thromboembolic events, bleeding complications, or mortality. The lack of follow‐up data prevents any assessment of the efficacy or safety of the observed antithrombotic strategies. Notably, the dataset originates from 2020. Given the rapid evolution of clinical guidelines and recent epidemiological shifts in AF management, these findings represent a specific temporal snapshot and may not fully reflect the most current clinical practices or the latest therapeutic trends. Our results should be interpreted with appropriate caution regarding their applicability to the present‐day landscape.

Second, since all participating centers are located in Shanghai, a major metropolitan area with advanced medical resources, the study may not adequately reflect practices in regions with different healthcare infrastructures, such as rural settings.

Third, because medication regimens were recorded only at discharge, we lack data on treatment duration, patient adherence, and subsequent therapy adjustments, which are essential to understanding the complete management trajectory. The appropriateness of DOAC dosing was described observationally based on discharge prescriptions rather than formally evaluated against guideline‐recommended criteria. This distinction is critical, as standardized dosing benchmarks for combined antithrombotic therapy are less established than those for AF monotherapy. Therefore, the observed prevalence of reduced dosing should be interpreted as a descriptive reflection of real‐world precautionary prescribing patterns, rather than a formal assessment of inappropriate clinical practice.

Furthermore, the absence of detailed procedural characteristics (e.g., left main disease and complex PCI) limited our ability to analyze how regimens were tailored to specific ischemic risk profiles. Lastly, although this study integrated data from 36 centers, intercenter clustering or variability was not formally modeled; thus, the potential influence of local institutional practices or physician preferences on treatment patterns cannot be entirely excluded.

## Author Contributions

**Jiwei Yu:** data curation, investigation, formal analysis, writing – original draft. **Shuolin Liu:** formal analysis, writing – original draft. **Run Du:** methodology, supervision, writing – review and editing. **Qinglan Tao:** data curation, formal analysis, visualization, writing – review and editing. **Jinzhou Zhu:** visualization, writing – review and editing. **Fenghua Ding:** conceptualization, methodology, supervision, project administration. **Ruiyan Zhang:** resources, conceptualization, supervision, project administration. **Zhengbin Zhu:** resources, supervision, project administration, writing – review and editing. Jiwei Yu, Shuolin Liu, Run Du, and Qinglan Tao contributed equally to this work and should be regarded as co‐first authors.

## Funding

This work was supported by the Science and Technology Commission of Shanghai Municipality (Grant Numbers: 24SF1904800 and 25SF1902203).

## Conflicts of Interest

The authors declare no conflicts of interest.

## Supporting information


**Supporting Information** Additional supporting information can be found online in the Supporting Information section. Table S1: Missing rates of study variables and management of the missingness. Table S2: Baseline characteristics of the study population, stratified by gender. The table presents a comprehensive comparison of demographic, clinical, echocardiographic, and treatment profile data between female (*N* = 385) and male (*N* = 921) patients. Table S3: Detailed breakdown of antithrombotic regimens and dosages for the subset of patients receiving oral anticoagulation therapy (*N* = 719), including data on vitamin K antagonist (VKA) control and rivaroxaban/dabigatran dosing strategies. These tables provide essential supporting details that underpin the baseline comparisons and treatment analyses discussed in the main text.

## Data Availability

The data that support the findings of this study are available from the corresponding author, Zhengbin Zhu, upon reasonable request.
