# Supplementary material for: Real‐World Antithrombotic Management of Nonvalvular Atrial Fibrillation in Patients Undergoing Percutaneous Coronary Intervention in China: A Cross‐Sectional Study
Source: Cardiovasc Ther. 2026 Jun 4;2026:5163410. doi: 10.1155/cdr/5163410 (PMC13238237; doi:10.1155/cdr/5163410)
Supplement: Supplementary file 1 — Supporting Information Additional supporting information can be found online in the Supporting Information section. Table S1: Missing rates of study variables and management of the missingness. Table S2: Baseline characteristics of the study population, stratified by gender. The table presents a comprehensive comparison of demographic, clinical, echocardiographic, and treatment profile data between female (N = 385) and male (N = 921) patients. Table S3: Detailed breakdown of antithrombotic regimens and dosages for the subset of patients receiving oral anticoagulation therapy (N = 719), including data on vitamin K antagonist (VKA) control and rivaroxaban/dabigatran dosing strategies. These tables provide essential supporting details that underpin the baseline comparisons and treatment analyses discussed in the main text. [file CDR-2026-5163410-s001.docx]

| **Variable** | **Missing, n(%),**  **(N=1306)** | **Management of missing data** |
| --- | --- | --- |
| BMI | 85 (6.5) | Imputed using multiple imputation method implemented by SPSS software |
| LVEF | 66 (5.0) |  |
| LA | 73 (5.6) |  |
| PASP | 94 (7.2) |  |
| **Table S1. Missing rates of study variables and management of the missingness***  *The following parameters are complete: Age, sex, AF type, CAD type, medical history, CHA_2_DS_2_-VASc score, HAS-BLED score, medical institution profile and antithrombotic regimens. | | |

| **Variable** | **Female**  **N=385** | **Male**  **N=921** | **p value** |
| --- | --- | --- | --- |
| **Demographic characteristics** |  |  |  |
| Age at diagnosis, median (IQR) | 76.74±8.19 | 71.86±8.84 | <0.001 |
| <65, n (%) | 30 (7.8%) | 175 (19.0%) | <0.001 |
| 65-74, n (%) | 119 (30.9%) | 396 (43.0%) |  |
| ≥75, n (%) | 236 (61.3%) | 350 (38.0%) |  |
| BMI, median (IQR) | 24.4±3.6 | 24.7±3.1 | 0.103 |
| **AF type, n (%)** |  |  |  |
| Paroxysmal atrial fibrillation | 215 (55.8%) | 512 (55.6%) | 0.933 |
| Persistent/ permanent atrial fibrillation | 170 (44.2%) | 409 (44.4%) |  |
| **CAD type, n (%)** |  |  |  |
| SAP | 84 (21.8%) | 242 (26.3%) | 0.042 |
| UAP | 192 (49.9%) | 396 (43.0%) |  |
| NSTEACS | 56 (14.5%) | 119 (12.9%) |  |
| STEMI | 53 (13.8%) | 164 (17.8%) |  |
| **Medical history, n (%)** |  |  |  |
| CHF | 169 (43.9%) | 379 (41.2%) | 0.359 |
| NYHA I | 5 (1.3%) | 12 (1.3%) | 0.729 |
| NYHA II | 86 (22.3%) | 200 (21.7%) |  |
| NYHA III | 67 (17.4%) | 135 (14.7%) |  |
| NYHA IV | 11 (2.9%) | 32 (3.5%) |  |
| Hypertension | 316 (82.1%) | 707 (76.8%) | 0.034 |
| Hyperlipemia | 44 (11.4%) | 77 (8.4%) | 0.081 |
| DM | 160 (43.6%) | 348 (37.8%) | 0.202 |
| Stroke | 83 (21.6%) | 180 (19.5%) | 0.408 |
| Ischemic | 83 (21.6%) | 168 (18.2%) | 0.082 |
| Hemorrhagic | 0 (0.0%) | 10 (1.1%) |  |
| Ischemic+hemorrhagic | 0 (0.0%) | 2 (0.2%) |  |
| Prior-MI | 90 (23.6%) | 275 (29.9%) | 0.021 |
| Prior-Bleeding | 7 (1.8%) | 29 (3.1%) | 0.181 |
| Renal disease (Cr >2.26mg/dL) | 22 (5.7%) | 62 (6.7%) | 0.501 |
| Current smoker | 15 (3.9%) | 381 (41.4%) | <0.001 |
| Current drinker | 45 (11.7%) | 138 (15.0%) | 0.118 |
| **Echocardiographic, median (IQR)** |  |  |  |
| LVEF | 58.3±12.5 | 55.3±13.3 | <0.001 |
| LA | 42.1±9.2 | 42.4±11.5 | 0.794 |
| PASP | 34.2±13.8 | 33.5±13.6 | 0.163 |
| **Clinical risk score** |  |  |  |
| **CHA_2_DS_2_-VASc score, mean (SD)** | 5.0±1.57 | 3.67±1.53 | <0.001 |
| 1, n (%) | 2 (0.5%) | 64 (6.9%) | <0.001 |
| 2, n (%) | 11 (2.9%) | 151 (16.4%) |  |
| 3, n (%) | 52 (13.5%) | 229 (24.9%) |  |
| 4, n (%) | 89 (23.1%) | 215 (23.3%) |  |
| 5, n (%) | 96 (24.9%) | 150 (16.3%) |  |
| 6, n (%) | 70 (18.2%) | 75 (8.1%) |  |
| 7, n (%) | 40 (10.4%) | 29 (3.1%) |  |
| 8, n (%) | 16 (4.2%) | 8 (0.9%) |  |
| 9, n (%) | 9 (2.3%) | 0 (0.0%) |  |
| **HAS-BLED score, mean (SD)** | 2.40±0.92 | 2.30±0.98 | 0.069 |
| 0, n (%) | 7 (28.0%) | 18 (2.0%) | 0.094 |
| 1, n (%) | 37 (9.6%) | 145 (15.7%) |  |
| 2, n (%) | 189 (49.1%) | 414 (45.0%) |  |
| 3, n (%) | 106 (27.5%) | 252 (27.4%) |  |
| 4, n (%) | 41 (10.6%) | 72 (7.8%) |  |
| 5, n (%) | 4 (1.0%) | 16 (1.7%) |  |
| 6, n (%) | 1 (0.3%) | 3 (0.3%) |  |
| 7, n (%) | 0 (0.0%) | 1 (0.1%) |  |
| **Medical Institution profile** |  |  |  |
| Treated in China AF Center, n (%) | 259 (67.3%) | 661 (71.8%) | 0.104 |
| PCI procedure Volume, median (IQR) | 1213 (729-1921) | 1213 (729-1921) | 0.135 |
| **Antithrombotic Regimens, n (%)** |  |  |  |
| OAC+DAPT | 78 (20.3%) | 236 (25.6%) | 0.019 |
| OAC+SAPT | 110 (28.6%) | 195 (21.2%) |  |
| DAPT | 172 (44.7%) | 412 (44.7%) |  |
| OAC | 25 (6.5%) | 75 (8.1%) |  |
| SAPT | 0 (0.0%) | 3 (0.3%) |  |
| **Table S2. Baseline characteristics of the study population, stratified by gender.**  ***** Comparisons were made between genders.  IQR, interquartile range; BMI, body mass index; AF, atrial fibrillation; CAD, coronary artery disease; SAP, stable angina pectoris; UAP, unstable angina pectoris; NSTE-ACS, non-ST-segment elevation acute coronary syndromes; STEMI, ST-segment elevation myocardial infarction; CHF, congestive heart failure; NYHA, New York Heart Association; DM, diabetes mellitus; Cr, creatinine; LVEF, left ventricular ejection fraction; LA, left atria; PASP, pulmonary arterial systolic pressure; SD, standard deviation; PCI, percutaneous coronary intervention; OAC, oral anticoagulants; DAPT, dual antiplatelet therapy; SAPT, single antiplatelet therapy. | | | |

| **Patients receiving anticoagulation therapy, n/total number (%)** | |
| --- | --- |
| VKA-based treatment | 87/719 (12.1%) |
| INR control |  |
| VKA+SAPT | 23/87 (26.4%) |
| INR <2 | 12/87 (13.8%) |
| INR 2-3 | 11/87 (12.6%) |
| VKA+DAPT, | 49/87 (56.3%) |
| INR <2 | 32/87 (36.8%) |
| INR 2-3 | 15/87 (17.2%) |
| INR>3 | 1/87 (1.1%) |
| VKA monotherapy | 15/87 (17.2%) |
| INR <2 | 2/87 (2.3%) |
| INR 2-3 | 13/87 (14.9%) |
| Rivaroxaban-based treatment | 552/719 (76.8%) |
| Rivaroxaban+SAPT | 247/552 (44.7%) |
| 2.5 mg bid/5 mg qd | 102/552 (18.5%) |
| 10 mg qd | 60/552 (10.9%) |
| 15 mg qd | 82/552 (14.9%) |
| 20 mg qd | 3/552 (0.5%) |
| Rivaroxaban+DAPT | 231/552 (41.8%) |
| 2.5 mg bid/5 mg qd | 146/552 (26.4%) |
| 10 mg qd | 48/552 (8.7%) |
| 15 mg qd | 34/552 (6.2%) |
| 20 mg qd | 3/552 (0.5%) |
| Rivaroxaban monotherapy | 74/552 (13.4%) |
| 2.5 mg bid/5 mg qd | 8/552 (1.4%) |
| 10 mg qd | 10/552 (1.8%) |
| 15 mg qd | 52/552 (9.4%) |
| 20 mg qd | 4/552 (0.7%) |
| Dabigatran-based treatment | 80/719 (11.1%) |
| Dabigatran 110 mg bid | 80/80 (100.0%) |
| **Table S3 Antithrombotic regimen details and dosing for patients receiving oral anticoagulation therapy.**  VKA, Vitamin K antagonist; SAPT, Single antiplatelet therapy; DAPT, Dual antiplatelet therapy; ASA, acetylsalicylic acid; INR, International normalized ratio; bid, twice daily; qd, once daily. | |
